# Electric vagal nerve stimulation inhibits inflammation and improves early postoperation cognitive dysfunction in aged rats

**DOI:** 10.1186/s12871-019-0885-5

**Published:** 2019-11-23

**Authors:** Jun Xiong, Huijun Wang, Yin Bao, Yuliang Guo, Yongxing Sun

**Affiliations:** 1 Department of Anesthesiology, Sanbo Brain Hospital, Capital Medical University, No. 50 Yikesong, Xiangshan, Haidian District, Beijing, 100093 China; 2 Department of Anesthesiology, Tongren Hospital, Capital Medical University, No. 1 Dongjiao Minxiang, Dongcheng District, Beijing, 100730 China

**Keywords:** Vagus nerve stimulation, Cognitive dysfunction, Inflammation, General anesthesia

## Abstract

**Background:**

This study aimed to evaluate effects of electric vagal nerve stimulation on early postoperation cognitive dysfunction in aged rats.

**Methods:**

A total of 33 male Sprague Dawley rats were selected and assigned randomly to three groups, control group (C, *n* = 10), splenectomy group (S, *n* = 10) and splenectomy+vagal nerve stimulation group (SV, *n* = 13). Behavior and memory of rats were evaluated by Open Field Test and Morris Water Maze. Levels of TNF-α, IL-6 and IL-10 in serum were measured by ELISA. The level of TNF-α protein in hippocampus was assessed by Western blotting. rt-PCR was used to detect mRNA expression of NF-κB in hippocampus.

**Results:**

During anesthesia/operation, vital life signs of rats were stable. In SV group, vagal nerve stimulation decreased heart rate lower than 10% of basic level and kept it at a stable range by regulating stimulation intensity. After stimulation stop, heart rate returned to the basic level again. This indicated that the model of vagal nerve stimulation was successful. Serum levels of TNF-α and IL-6 increased by the operation/anesthesia, but they decreased with vagal nerve stimulation (all *P* < 0.05). TNF-α protein and mRNA expression of NF-κB in hippocampus were also eliminated by vagal nerve stimulation compared to S group (*P* < 0.05). Results of Morris Water Maze showed escape latency of postoperation in S group was significantly longer than C group (*P* < 0.05), and times of crossing platform in S group was lower than that of C group (*P* < 0.05). Although escape latency of postopration in SV group was shorter than that of S group, there was no significant difference between two groups. Meanwhile there were no significant differences of behavior test in Open Field test between three groups, although vagal nerve stimulation improved partly active explore behavior compared to S group.

**Conclusion:**

The inflammation caused by operation and general anesthesia was an important reason of early postoperation cognitive dysfunction, and electric vagal nerve stimulation could inhibit the inflammation. Meanwhile, vagal nerve stimulation could ameliorate early postoperation cognitive dysfunction partly, but its protective effects were not enough and should be studied and improved in future.

## Background

Postoperation cognitive dysfunction (POCD) is a severe complication of surgery and general anesthesia. Fourteen percent of patients have been found cognitive decline and confusion after surgery with anesthesia [1, 2]. POCD is frequent in patients older than 60-year undergoing major non-cardiac surgery, which increases both morbidity and mortality [3]. These cognitive impairments are related to language comprehension, attention, social integration and short term memory. Also, POCD may extent recovery process and hospital stay, and diminish quality of patients’ life [4]. There are many risks developing postoperative cognitive decline in elderly patients, such as increased age, longer time in surgery, longer stay in an intensive care unit and mechanical ventilation time [2]. Although POCD is not rare, its underlying pathogenic mechanisms have not been known completely.

The systemic inflammation has been identified as an important process for occurrence and development of POCD [4, 5]. Not only surgical trauma, but also inhaled anesthetics could produce systemic inflammatory response, which leads to blood-brain barrier disruption, neuro-inflammation and cognitive dysfunction [6, 7]. So inhibiting this systemic inflammation might be a potential strategy preventing and/or treating cognitive dysfunction.

Vagal nerve has been identified to link to inflammatory response, its activity could suppress TNF-α and other pro-inflammatory cytokines. This anti-inflammation arc has been well-known as cholinergic anti-inflammation pathway [8]. Vagal nerve regulates inflammatory response through hypothalamic-pituitary-adrenal axis, release of cortisol and vagovagal reflex [9]. Anti-inflammatory effects of vagal nerve stimulation (VNS) were firstly researched by Borovikova and colleague [10]. They found not only acetylcholine, as vagal neurotransmitter, inhibited release of inflammatory cytokines in lipopolysaccharide stimulated human macrophage cultures, but also direct VNS could reduce systemic inflammatory response and prevent progress of infectious shock by attenuating inflammatory cytokines synthesis. Following their works, the anti-inflammation mechanisms of VNS have been researched systemically. Although the whole mechanisms and the signaling pathway have been unknown completely, wider range of inflammatory disorders might be ameliorated by VNS [11].

The aim of this study, firstly, was to demonstrate whether surgery and general anesthesia could induce systemic- and neuro-inflammatory response and impair cognitive function in elder rats. Secondly, it was to evaluate effects of VNS improving cognitive dysfunction by inhibiting the inflammatory response.

## Methods

### Experimental animals

A total of 33 male Sprague Dawley (SD) rats were obtained from Beijing Vital River Laboratory Animal Technology Co. Ltd. (Beijing, 100,012, China. \ (Jing)2012–0001.) and maintained in suitable rooms with controlled conditions of temperature at 22 ± 1 °C, 40 ± 10% relative humidity and light-dark cycles (12 h- 12 h, light onset at 7:00). Five rats were housed in one cage. During this experiment, standard food and drink water were available to the animals ad libitum. This study was approved by the institution ethical committee for animal care and use, Sanbo Brain Hospital, Capital Medical University. All efforts were made to minimize the number of animals used and their suffering. Experiments were performed during day, always at the same time, to avoid circadian variations. After finishing behavior tests, all animals were humanely euthanized using intraperitoneal injection of 3% sodium pentobarbital (50 mg/kg) and rapid decapitation. The process was as soon as possible to avoid interference on the results of experiment.

### Experiment protocol

**Fig. 1:**
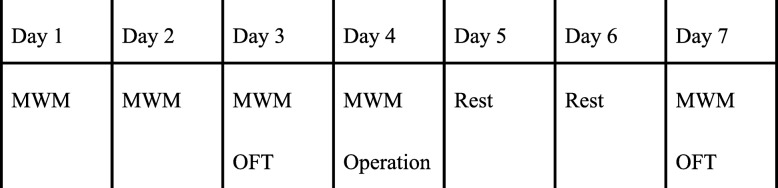
The experiment protocol. MWM: Morris water maze. OFT: Open field test

A random number generator was used to allocate the rats into different groups: control group (*n* = 10, C group, 577.00 ± 47.53 g) that received no surgery and anesthesia, splenectomy group (*n* = 10, S group, 577.60 ± 33.73 g) that was isolated cervical vagal nerve without stimulation, and splenectomy+VNS group (*n* = 13, SV group, 571.15 ± 50.63 g). Then all rats were conducted with Morris Water Maze (MWM) train (day 1–4) and test on day 7, Open Field Test (OFT) train on day 3 and test on day 7, splenectomy was carried on the day 4. Finishing behavior tests, all animals were decapitated for tissue preparation on day 7 (Fig. 1).

### Electric vagal nerve stimulation

Rats were fixed in a cage and anesthetized with 1% propofol (Fresenius Kabi AB. Rapsgatan 7, 751 74 Uppsala. Sweden. Serial number: 10MC2871.) 80 mg/Kg intraperitoneally, after losing righting reflex, their tail vein was cathetered with 24-gauge catheter infusion set (Tuoren Medical Device Co., Ltd. Henan, 453,401, China.) for continuous propofol infusion [12]. During the experiment, rats were allowed to breathe pure oxygen through a tube filled with oxygen continuously, which fixed in front of their nose in order to prevent hypoxia. After 10 min stabilization, heart rate was recorded as basic line. Thus, neck hair shaving and skin cleaning, aseptic technique was used to make a ventral midline incision in neck skin. Then skin and muscles were retracted. Because right vagal nerve primarily innervates atria and sinoatrial node, and these stimulation may induce significant change in cardiac rhythm. At the same time, right VNS produce smaller cardiorespiratory response, so right vagal nerve was applied [13, 14]. Isolating right cervical vagal nerve and common carotid artery bundle, a 1.5 mm diameter silver bipolar cuff electrode was gently wrapped around the nerve bundle and fixed to the sternocleidomastoid muscle. Then the electrode was connected to stimulator (BL-820 Biological signal acquisition and processing system. Chengdu Techman Software Co. Ltd. Sichuan, Chengdu, 610,100, China). The basic stimulation parameters were adapted to the threshold of individual animal and included 2 V, 10 Hz and 1 ms, but these parameters were regulated constantly to preserve heart rate lower than 10% of basic line [14–17].

### Splenectomy

After 30 min of VNS, splenectomy began with a small lateral peritoneal incision. Using 3.0 silk thread to dissociate and ligate spleen artery and vein at hilum of spleen, spleen was removed at the root of far end of spleen pedicle. The completely removed spleen was examined to ensure that no residual spleen was left. Then the incision was closed, covering it with sterile activated-iodine gauze, and securing it with adhesive tape. The operation time was within 60 min.

### Behavioral tests

#### Open field test

OFT was applied in an apparatus, which was made of brown plywood, surface area was 50 × 50 cm, surrounded by 50 cm high walls. The floor was divided by black line into 25 rectangles. Rats were allowed to freely move in this apparatus for 5 min. The movement of individual animal in the arena was automatically tracked by AVTAS ver5.0 animal video analysis system (AniLab Software & Istruments Co., Ltd. Ningbo, China.) The number of crossing and rearing activities by each rat during 5 min was used to assess rats’ active explore behavior. After every test, the apparatus was cleaned with 5% ethanol.

#### Morris water maze

MWM was used to evaluate spatial reference learning and memory. A circle pool (150 cm in diameter and 80 cm deep) was filled with water and divided into four quadrants. An escape platform, which 40 cm in height and 15 cm in diameter, was submerged by 2 cm under water surface and conserved to the center of northwest (NW) quadrant of this pool. Water was maintained at temperature of 23 ± 2 °C. The evaluation consisted of four training days of five consecutive trails per day, the last trail of each day was accepted. Rats were randomly introduced in this tank from different quadrants facing wall to find the escape platform in 60 s. If the rats did not find the platform within 60s in the first trial, they were gently guided to the platform to remain for 30s. Then the rats were removed from the tank. This procedure ensured animals to retain visual-spatial information during trail. The movement of individual rat was automatically tracked by AVTAS ver5.0 animal video analysis system (AniLab Software & Istruments Co., Ltd. Ningbo, China.) On the last day of the test after operation, rats were assessed in this tank without the platform. The time to find the platform, the times of passing through the platform location, and the duration of time in the quarter of platform were counted.

#### Assessment of TNF-α, IL-6, IL-10 in serum

Levels of serum TNF-α, IL-6, IL-10 were measured by Enzyme-Linked Immunosorbent Assay (ELISA). Aliquots containing 100ul of serum were placed into wells of ELISA plates and the plate was incubated at 37 °C for 2 h. Primary antibodies were rabbit monoclonal antibodies to TNF-α (Huamei, Wuhan. Number CSB-E11987r, 1:100 dilution), IL-6 (Huamei, Wuhan. Number CSB-E04640r, 1:100 dilution), IL-10 (Huamei, Wuhan. Number CSB-E04595r, 1:100 dilution). The primary antibodies were added to each wells and incubated at 37 °C for 1 h. After three washes with PBS-Tween 20 (0.1%), a horseradish-peroxidase-conjugated goat anti-rabbit IgG was added into the wells and incubated for 1 h at 37 °C. The antibody-antigen complex was revealed by addition of 100ul of 2,2′-azinobis (3-ethylbenzthiazoline-6-sulfonic acid) (ABTS) containing 0.3% H_2_O_2_ into each well. After 15 min, optical density (OD) was determined using Bio Tek Epoch (Bio Tek Instruments, Inc. China. Beijing, 100,025, China.) at 450 nm. All samples were processed under the same experimental conditions and time.

#### TNF-α protein in hippocampus

The proteins were extracted from hippocampus and their concentration was determined by Bicinchoninic Acid Kit (Catalog BCA02, Dingguo Changsheng Biology Technology LTD, Beijing, China). 30μg protein samples were separated by SDS-PAGE, followed by semi-dry transfer onto a PVDF membrane. The membrane was blocked in 5% non-fat dry milk. Further, membranes were incubated with rat monoclonal primary antibody, anti-TNF-α and anti-tubulin, for overnight at 4 °C. This was followed by triple washing with 0.1% TBST and incubation with HRP labeled secondary antibodies for 2 h at RT. Immunoreactive bands were detected by ECL and Western blot detection system using Quantity one (Bio-Rad Laboratories. Inc. Hercules, CA, USA). These steps were repeated triply in order to calculate mean value. Expressions of each interest protein were calculated after normalizing the interest protein with endogenous control tubulin in the same sample.

#### NF-κB in hippocampus expression

Total RNA was isolated from 100 mg of hippocampal cortex using 1 ml of TRIzol (Invitrogen Corporation; Carlsbad, CA, USA) and then RNA was treated with RNase-free DNase I and quantified using Q5000 Spectrophotometer (Quawell Technology, Inc. San Jose, CA, USA). cDNA was obtained from 5μg of total RNA using 2ul of ReverTra Ace reverse transcriptase kit (Catalog TRT-101, TOYOBO STC (Shanghai) CO., LTD, Shanghai, China), 2ul of Oligo dT 50um, 2ul of dNTP mix 10 mM, and water grade molecular biology to 20ul. Retrotranscription conditions were 30 °C for 10 min, followed 42 °C for 60 min and 99 °C for 5 min, then 4 °C for 5 min. Finally, the cDNA was stored at − 20 °C.

cDNA was used to amplify each gene using Sybr Green I (Catalog GG1301–50, Gen-View Scientific Inc. Florida, USA). The amplification reactions contained 1 ul of respective SybrGreen I, 12.5ul of Mix (Catalog PER012–1, Dingguo Changsheng Biology Technology LTD, Beijing, China), and 1ul of cDNA in a final volume of 25ul. The conditions were for qPCR were 3 min for pre-denaturation at 95 °C, followed by 35 cycles of amplification of 30s denaturation at 94 °C, 30s annealing at 60 °C and 30s extending at 72 °C. The last extending step was 10 min at 72 °C. Rat GAPDH was used as internal control gene for normalization. The amplification assays were made using ABI PRISM 7700 Sequence Detection System (ThermoFisher Scientific (China) LTD, Shanghai, China). Primer pairs for quantitative real-time PCR were as follows: NF-κB, 5′-AACCTGGGAATACTTCATGTGACTAA-3′(sense) and 5′-GCACCAGAAGTCCAGGATTATAGC-3′ (anti-sense), GADPH, 5′- GCTGAGTATGTCGTGGACTC-3′ (sense) and 5′- TTGGTGGTGCAGGATGCATT-3′ (anti-sense). The steps were repeated triply in order to calculate mean value. The 2^-ΔΔCt^ analyses were applied to calculate the relative transcript levels expressed as fold change for gene expression [18].

### Statistical analysis

SPSS 16.0 for Windows software package (SPSS, Inc., Chicago, IL, USA.) was used to perform statistical analysis. Quantitative data were expressed as mean ± standard deviation (^−^*x* ± *s*). Normal distribution of data was check by Kolmogorov-Smirnov analysis. One-way ANOVA Post Hoc Multiple-Comparisons was used for multi-group comparisons of means. When homogeneity of variances occurred, L-S-D was used to compare between groups. When variances were heterogeneous, Dunnett T3 was used to compare between groups. Two-way repeated-measured ANOVA was use to analysis the data of escape latency. The mean difference was significant at 0.05 level.

## Results

**Fig. 2:**
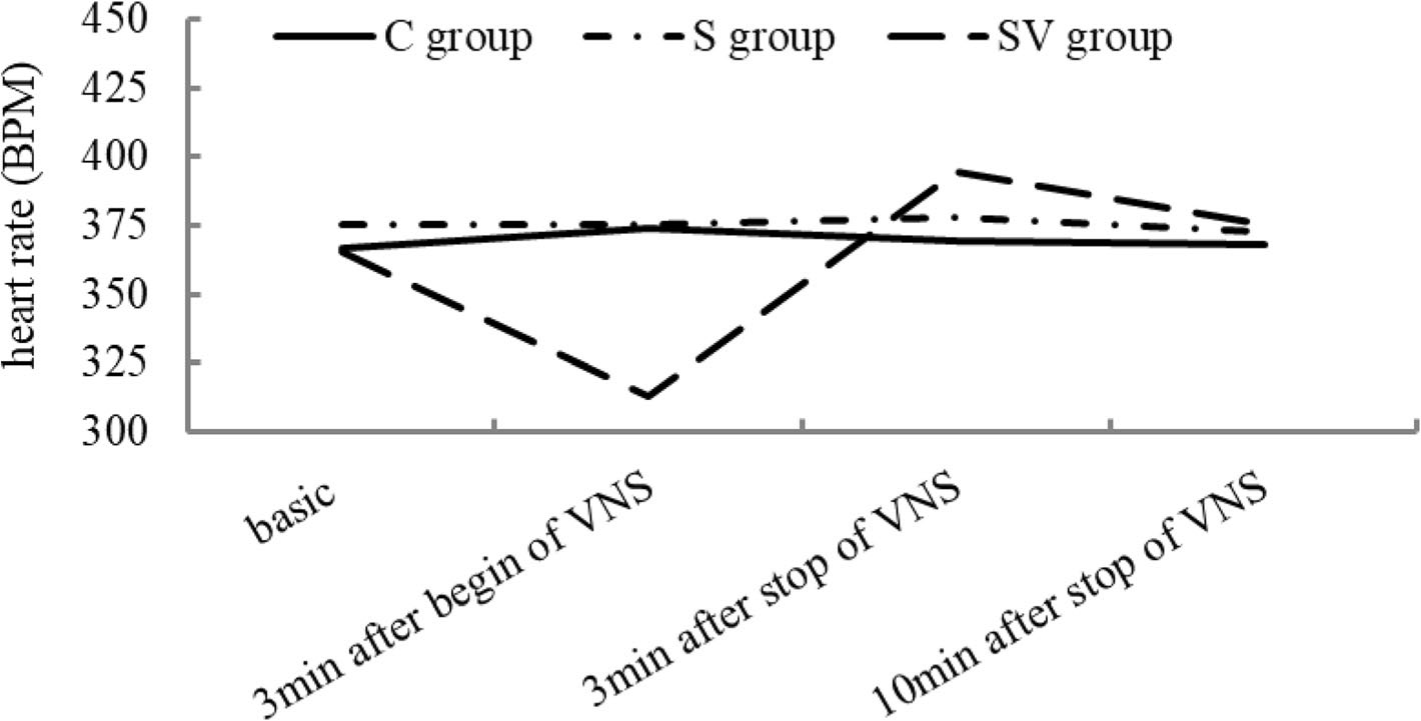
The basic HR was no significant difference between three groups. At 3 min after begin of VNS, the HR in SV group was lower than the other groups. And at 3 min after stop of VNS, the HR of SV group was slightly increased, compared to the other groups. Ten min after stopping VNS, the HR of SV group return to the basic level, no difference of HR between three groups

There were no significant different between three groups on body weight (*F* = 0.073, *P* = 0.930) and basic heart rate (*F* = 1.163, *P* = 0.326) (Table 1). Three minutes after beginning of VNS, heart rates of SV group (312.85 ± 27.52) was reduced significantly, compared to C group (373.90 ± 21.40) and S group (375.30 ± 16.26) (*F* = 28.928, *P* < 0.01). Three minutes after stopping VNS, heart rates of SV group (394.85 ± 32.19) rose up, compared to C group (369.60 ± 21.46) (*P* = 0.021). Till to ten minute after stopping VNS, heart rates were not significantly different between three groups (*F* = 0.230, *P* = 0.796), and recovered to the basic level (Fig. 2).

**Table 1:** The body weight and basic heart rate of three groups

Groups	Body weight	Basic heart rate
C group	577.00 ± 47.53	366.60 ± 15.12
S group	577.60 ± 33.73	375.10 ± 14.39
SV group	571.15 ± 50.63	365.38 ± 17.73
*F*	0.073	1.163
*P*	0.930	0.326

### The level of inflammatory cytokines in serum

**Fig. 3:**
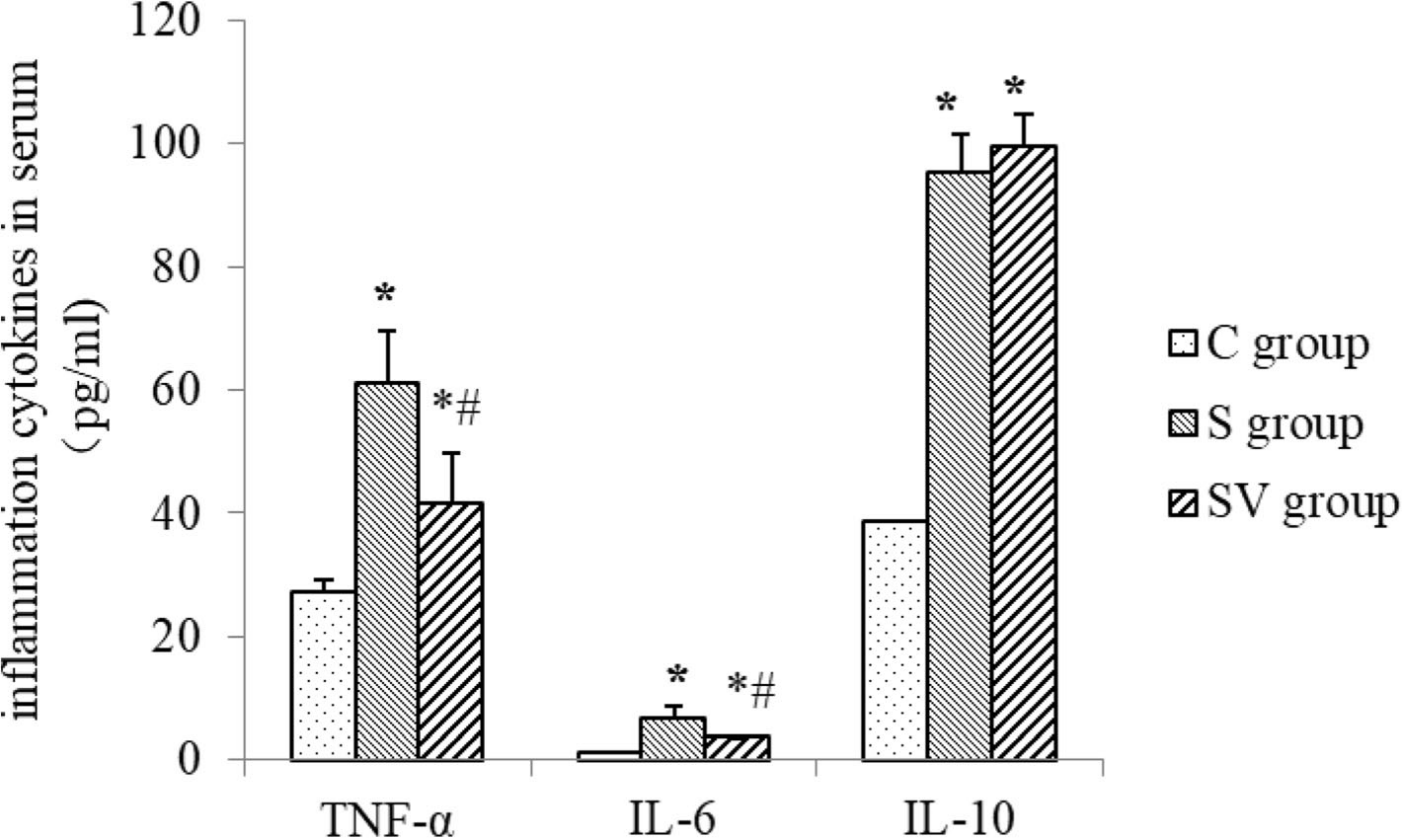
TNF-α, IL-6 and IL-10 in serum. Compared to the C group, TNF-α, IL-6 and IL-10 in the S and SV groups were significantly increased, *P* < 0.05. VNS could reduce TNF-α and IL-6 in serum significantly, compared to the S group, *P* < 0.05. VNS did slightly raised IL-10 in serum, but there was no significant difference between both of them. *P < 0.05, comparing to the C group, #*P* < 0.05 comparing to the SV group

TNF-α level was increased by surgery and general anesthesia in S group (61.028 ± 8.642 pg/ml, *P* < 0.001, Dunnett T3) and SV group (41.609 ± 8.249 pg/ml, *P* < 0.001, Dunnett T3), compared to C group (27.180 ± 2.038 pg/ml). And it could be reduced by VNS, because TNF-α in SV group was significant lower than that in S group (*P* < 0.001, Dunnett T3). IL-6 level was similar to TNF-α. Compared to C group (1.278 ± 0.258 pg/ml), IL-6 in S group (6.789 ± 1.827 pg/ml) and SV group (3.623 ± 0.685 pg/ml) were increased significantly (*P* < 0.001, Dunnett T3). But VNS decreased IL-6 in SV group compared to that of S group (*P* < 0.001, Dunnett T3). Anti-inflammatory cytokine IL-10 was higher in S group (95.351 ± 6.236 pg/ml) and SV group (99.387 ± 5.236 pg/ml) caused by splenectomy and general anesthesia, compared to C group (38.587 ± 0.645 pg/ml, *P* < 0.001, Dunnett T3). However, there was no significant difference between IL-10 of S and SV group (*P* = 0.302, Dunnett T3) (Fig. 3).

### The TNF-α level in the hippocampus

**Fig. 4:**
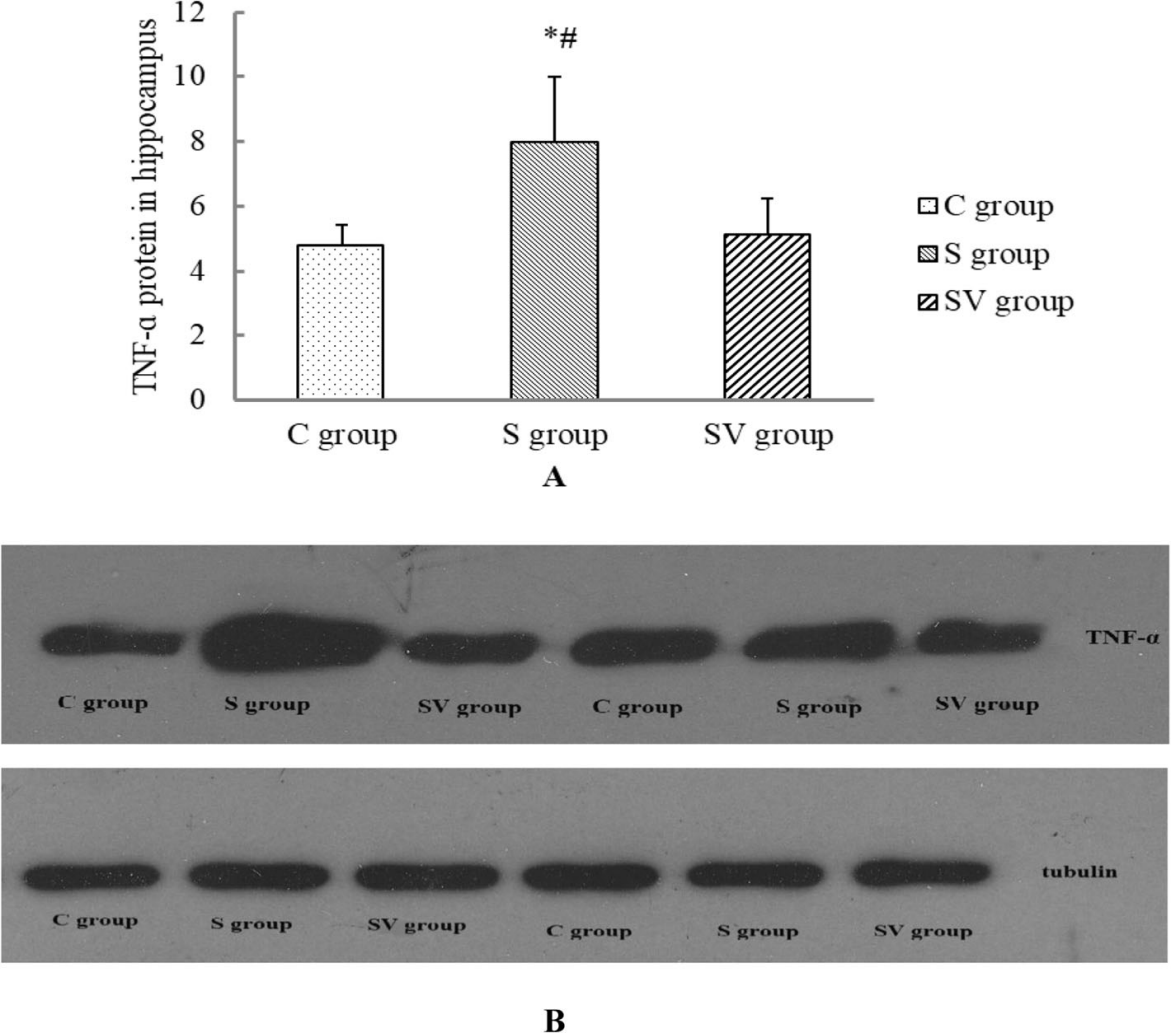
The TNF-α protein in hippocampus. **a** TNF-α protein in hippocampus was higher than that of other groups, *P* < 0.05. But TNF-α of the C and SV group were similar. *P < 0.05, comparing to the C group, #*P* < 0.05 comparing to the SV group (**b**)

As internal reference protein, tubulin level in three groups was no significant difference (*F* = 0.036, *P* = 0.965). Normalization with tubulin, TNF-α of three groups was significant difference (*F* = 10.018, *P* = 0.002).TNF-α in S group (8.00 ± 1.99) was significantly higher than that in C group (4.78 ± 0.63, Dunnett T3, *P* = 0.025) and SV group (5.13 ± 1.11, Dunnett T3, *P* = 0.043). But TNF-α protein of C and SV groups was similar (Dunnett T3, *P* = 0.872) (Fig. 4).

### The NF-κB gene expression in the hippocampus

**Fig. 5:**
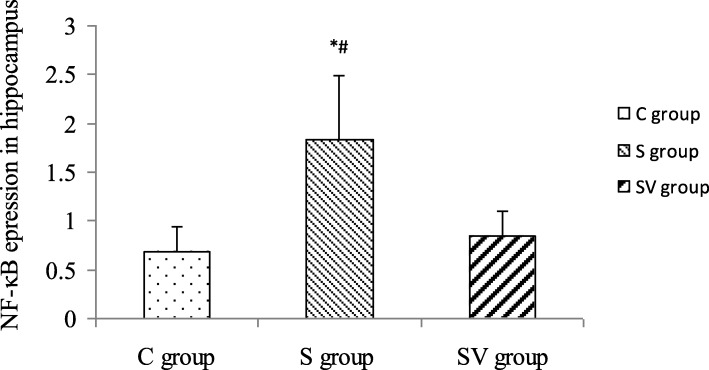
The NF-κB gene expression in hippocampus. The NF-κB expression of the S group was increased significantly compared to the C and SV groups. There was similar expression between the C and SV groups

After normalization with endogenous control GAPDH, NF-κB expression in three groups was significantly different (*F* = 12.648, *P* = 0.001, ANOVA). NF-κB gene of S group (1.839 ± 0.652) was increased significantly than that of C group (0.685 ± 0.253, *P* < 0.001, LSD) and SV group (0.849 ± 0.258, *P* = 0.001, LSD). And there was no significant difference of NF-κB gene expression between C and SV group (*P* = 0.518, LSD) (Fig. 5).

### Locomotor and exploratory activities

**Fig. 6:**
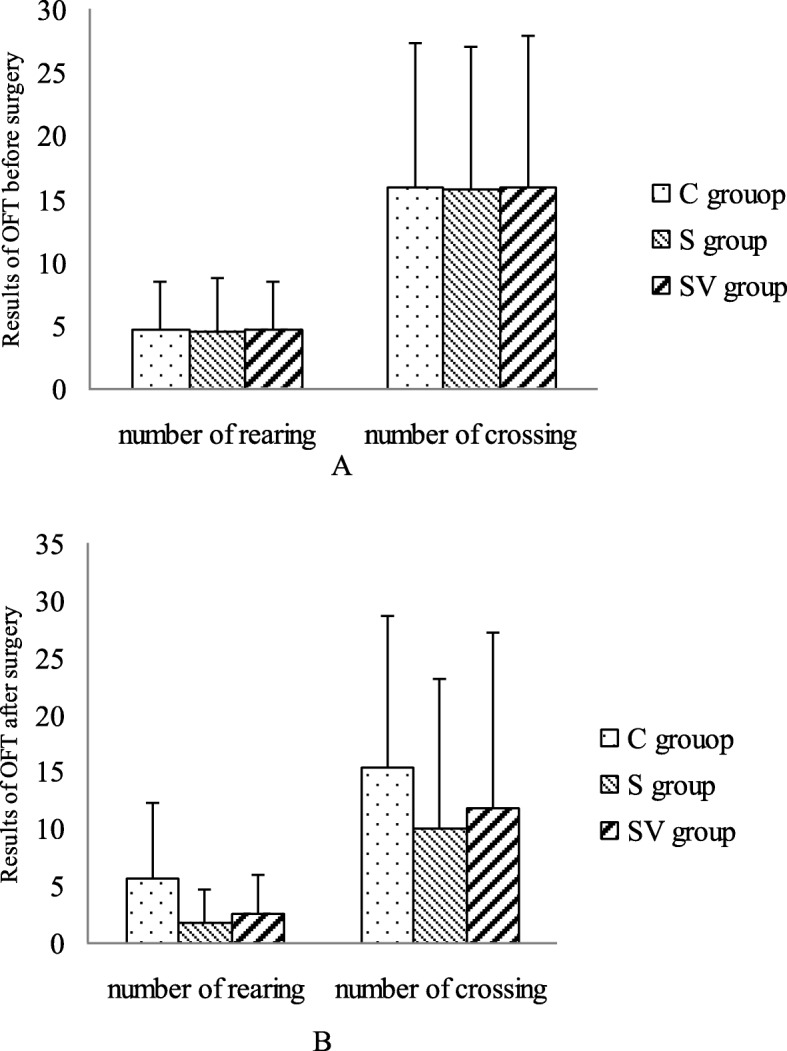
The results of Open Field Test. **a** Before the surgery, the numbers of crossing and the number of rearing were similar in three groups. **b** After the surgery, both of crossing and rearing number were no significant difference between three groups. But comparing to the S group, the number of crossing and rearing in the SV group increased

OPT was used to evaluate rat crossing and rearing activities, before and after splenecotmy respectively. Before the surgery, there were no significant different on the number of crossing (*F* = 0, *P* = 0.995) and rearing between three groups (*F* = 0.003, *P* = 0.966). After the operation, the number of crossing (*F* = 0.302, *P* = 0.587) and the number of rearing (*F* = 2.040, *P* = 0.148) were also similar in three groups, but the number of crossing (11.85 ± 15.44) and rearing (2.62 ± 3.30) in SV group were higher than the number of crossing (10.00 ± 13.19) and rearing (1.80 ± 2.82) in S group (Fig. 6).

### Effects of VNS on the MWM test

**Fig. 7:**
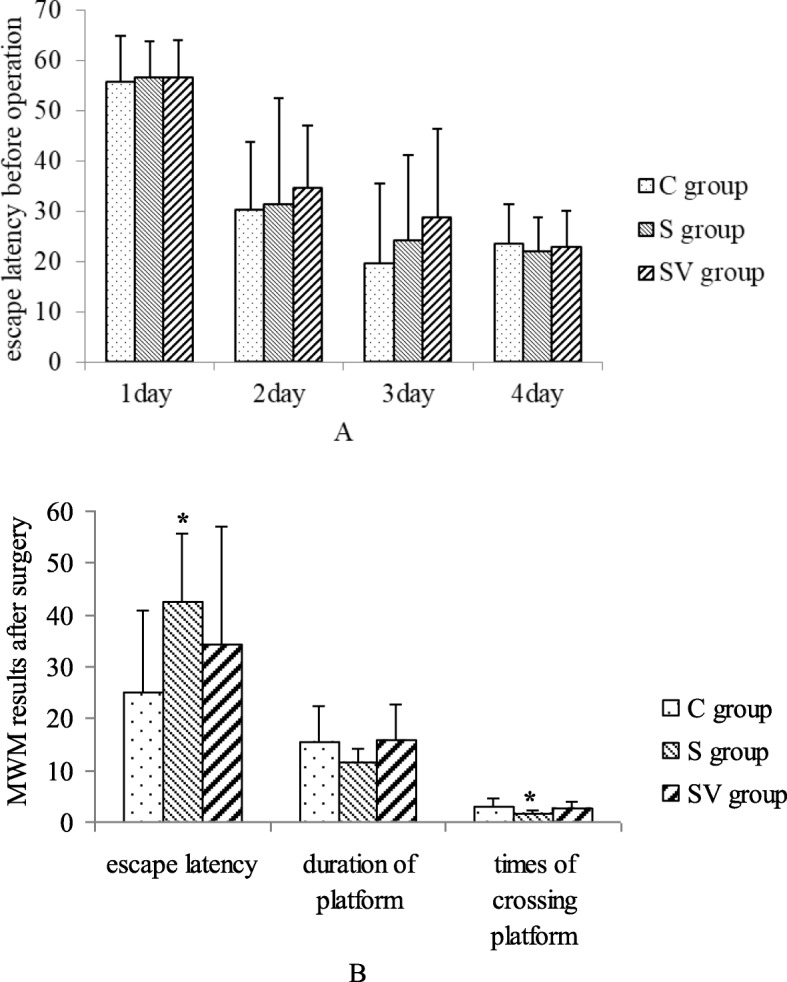
**a** Before the surgery on the continuous training days, the escape latency of three groups decreased day by day. There was no interaction between time and different intervention. Each day, the escape latency were no significant difference between three groups. **b** After the surgery, the escape latency and times of crossing platform in the S group were significantly different with these of the C group. The escape latency, duration of platform and times of crossing platform were similar in the C and SV groups. Although these parameters relating to study and memory were ameliorated by VNS, the difference was not significant

As shown in (Fig. 7), two-way repeated-measures ANOVA revealed that escape latency of three groups reduced over the 4 days training period before the operation (*F* = 103.062, *P* < 0.01), and there was no interaction between days, splenecotmy and VNS (*F* = 0.498, *P* = 0.807). Also escape latency of each day was no significant difference between three groups during 4 days’ training (*F* = 1.715, *P* = 0.208).

After the operation, S group had a longer escape latency (42.45 ± 13.23) than C group (25.07 ± 15.66, *P* = 0.045, Dunnett T3). And escape latency of C and SV group (34.37 ± 22.57, *P* = 0.578) was not significantly different. So escape latency was reduced by VNS compared to S group, but the difference was not significant. Results of times of crossing platform after the operation were similar to the results of escape latency. The rats of S group (1.6 ± 0.8) crossed few times than those of C group (2.9 ± 1.9, *P* = 0.046, LSD). And there was no significant difference between the times of crossing of C and SV groups (2.5 ± 1.3, *P* = 0.543, LSD). The times of crossing platform of SV group were more than that of S group, but the difference was not significant. After the operation, the duration of time spent in target quadrant was no significant difference between three groups (*F* = 1.751, *P* = 0.191). However, the time spending on the platform in S group (11.49 ± 2.58) was shorter than that of C group (15.54 ± 6.72) and SV group (15.73 ± 6.88).

## Discussion

Our results showed that surgery and general anesthesia could produce damage to memory and study capability, and aggravate POCD in elderly rats early after surgery and general anesthesia, because they induced systemic- and neuro-inflammation [19]. Although improvement of cognitive function was not significantly, electrical VNS did reduce POCD in some degree. The underlying mechanism might be related to inhibition of the inflammatory response caused by surgery and general anesthesia.

The inflammation caused by surgery and general anesthesia is one of most important reasons of POCD. There is strong evidence to suggest that acute inflammation affects and exacerbates cognitive function or cause delirium, one kind of clinical important postoperative complication. And ongoing inflammation might constantly impair cognitive function after surgery and anesthesia. Thus, inhibition or resolution of the inflammation is an important prerequisite for improvement of cognition [20]. In the present study, surgery and general anesthesia increased pro-inflammatory cytokines, TNF-α and IL-6. Meanwhile, IL-10, a kind of anti-inflammatory cytokine, was also upgraded. These changes demonstrated that acute systemic inflammation was induced via activation of innate immune system.

As an initiating medium of systemic inflammation, TNF-α activates and amplifies inflammation cascade. In addition, TNF-α can do damage directly to blood brain barrier and induce inflammatory cells infiltration in hippocampus [21]. Besides, NF-κB is an essential transcription factor to regulate inflammation and innate immunity genes expression. Its activation eventually increases level of pro-inflammatory cytokines in brain, such as TNF-α and IL-6, which subsequently cause cognitive impairment [22]. So NF-κB has been known as a potential therapeutic target, inhibition of NF-κB might improve cognitive dysfunction caused by sevoflurane anesthesia [23]. In our study, increase of pro-inflammation cytokines in serum and higher expression of TNF-α and NF-κB in hippocampus provided directly evidences that surgery and general anesthesia might induce acute inflammation, which took place systemically and locally, and lead to cognitive damage early after surgery and general anesthesia.

As to why right vagal nerve was stimulated in our study, this was because heart rate changed obviously when it was stimulated [13]. In other words, the change of heart rate is not only the evidence that VNS is successful, but also is the baseline for regulating stimulation parameters, such as stimulation intensity and frequency. In the present study, 3 min after onset of stimulation, heart rate reduced to about 85% of basic heart rate, and 10 min after stop of stimulation, heart rate increased to basic level. During the operation, continuous VNS kept heart rate at this level, till the operation was finished. In addition, right VNS might produce relatively slighter interruption to circulation and respiration than left VNS [14].

The more important is that vagal nerve plays key role in inflammation regulation, which is known as cholinergic anti-inflammation pathway. The vagal nerve efferent fibers release acetylcholine, which binding with special receptor on macrophages and inhibit production and release of TNF-α [24]. In our study, this effects of VNS were certified that VNS could decrease not only levels of pro-inflammatory cytokines in serum, such as TNF-α and IL-6, also TNF-α protein and transcription factor NF-κB in hippocampus were downgraded by VNS. As to the level of IL-10, although VNS could increase it slightly, there was no significant difference between S and SV groups. We inferred that anti-inflammation effects of vagal nerve focus on inhibition of pro-inflammatory cytokines, not upgrade of anti-inflammatory cytokines. So there was no significant change of IL-10.

Recently, Huffman and colleague reported that VNS could ameliorate cognitive response and decrease systemic and brain inflammation induced by lipopolysaccharide endotoxemia. In their study TNF-α was significantly inhibited by VNS [25], which was similar to results of our research. Besides effects of inhibiting inflammatory response, cholinergic system might regulate hippocampus function and memory, so it is not impossible to prevent or ameliorate POCD by VNS [26].

OFT is used to evaluate spontaneous activity and anxiety-like behaviors. In this study, results of OFT demonstrated that spontaneous movement in three groups were similar after the operation. Although VNS could ameliorate crossing and rearing movement, a kind of actively explore behaviors [27], there were no significant difference between three group. VNS was reported to increase score of OFT in depression model of rat and antagonized depressive status [28]. The results in the present study were not contradictory with the previous study, however these effects of VNS in our study was not significant statistically.

After the operation and general anesthesia, learning and memory function of rats were damaged, as indicated by increase of escape latency, and decrease of times of crossing platform and time spending on target region. VNS, as a kind of treatment, in our study, it did decrease escape latency, lengthen time spending on target quarter, and increase times of crossing platform, when compared to S group. These results were consistent with previous study which reported VNS ameliorated cognitive function [29]. However, in this study, these effects of VNS were not enough to produce protection against POCD completely.

Above results of OFT and MWM tests provided evidences that POCD could occur early after surgery, and inflammation caused by surgery and general anesthesia played more important role to induce POCD. These results were similar to recent studies [30, 31]. However, the detail mechanisms of POCD have not completely elucidated. For example, systemic inflammation, neuroinflammatin, cerebral microemboli and hypotension, all of these may cause POCD. In other words, any pathogenies could cause POCD as long as they interrupt central nerve system metabolic status and its homeostasis [32]. As well known, inflammation is one relatively important pathogeny for POCD, but it is not the only. Thus, we could explain the results of our study. Because VNS could inhibit the inflammation induced by surgery and general anesthesia, which demonstrated by lower level of pro-inflammation cytokines in serum and pro-inflammation protein and transcription factor NF-κB in hippocampus. However, there might be other pathogenies to promote the development of POCD. So VNS could ameliorate learning, memory function and actively explore movement in some degree, but its protection was not sufficient.

Our study indicated that surgery and general anesthesia could induce systemic and local inflammation. At the same time, cognitive function of rats was damaged. VNS might inhibit the inflammation to produce protective effect against POCD in some degree. However, this protection of VNS was insufficient. Maybe combination of VNS with other therapies might provide better clinical effects, this need to be researched in future.

### The limitation of this study

There were some limitations in our study. NF-κB is a key transcription factor to modulate inflammatory responses via regulating expression of pro-inflammation mediators. It is regulated by its inhibitor, IκB. NF-κB will be released by degradation of IκB. Then it enters nucleus and activates transcription of multiple inflammatory response genes by interacting with κB elements in promoter region. Thus, increased NF-κB activation is considered as an important pathogenic factor in many inflammatory disorders. In our study, we only tested total NF-κB expression. If the expression of IκB in hippocampus was tested, the more information relative with central nerve systemic inflammatory response would be demonstrated.

## Data Availability

The data of this article is available from the corresponding author. The email address of the corresponding author is B2008194@126.com.

## Abbreviations

ELISA: Enzyme-linked immunosorbent assay
MWM: Morris water maze
OFT: Open field test
POCD: Postoperation cognitive dysfunction
VNS: Vagal nerve stimulation

## References

[1] Xiong B, Shi Q, Fang H (2016). Dexmedetomidine alleviates postoperative cognitive dysfunction by inhibiting neuron excitation in aged rats. Am J Transl Res.

[2] Norkienė I, Samalavičius R, Misiūrienė I, Paulauskienė K, Budrys V, Ivaškevičius J (2010). Incidence and risk factors for early postoperative cognitive decline after coronary artery bypass grafting. Medicina (Kaunas).

[3] Besch G, Vettoretti L, Claveau M, Boichut N, Mahr N, Bouhake Y, Liu N, Chazot T, Samain E, Pili-Floury S (2018). Early post-operative cognitive dysfunction after closed-loop versus manual target controlled-infusion of propofol and remifentanil in patients undergoing elective major non-cardiac surgery: Protocol of the randomized controlled single-blind POCD-ELA trial. Medicine (Baltimore).

[4] Zhu H, Liu W, Fang H (2018). Inflammation caused by peripheral immune cells across into injured mouse blood brain barrier can worsen postoperative cognitive dysfunction induced by isoflurane. BMC Cell Biol.

[5] Zhang M, Zhang YH, Fu HQ, Zhang QM, Wang TL (2018). Ulinastatin May Significantly Improve Postoperative Cognitive Function of Elderly Patients Undergoing Spinal Surgery by Reducing the Translocation of Lipopolysaccharide and Systemic Inflammation. Front Pharmacol.

[6] Cao Y, Li Z, Ma L, Ni C, Li L, Yang N, Shi C, Guo X (2018). Isoflurane‑induced postoperative cognitive dysfunction is mediated by hypoxia‑inducible factor‑1α‑dependent neuroinflammation in aged rats. Mol Med Rep.

[7] Danielson M, Reinsfelt B, Westerlind A, Zetterberg H, Blennow K, Ricksten SE (2018). Effects of methylprednisolone on blood-brain barrier and cerebral inflammation in cardiac surgery-a randomized trial. J Neuroinflammation.

[8] Zila I, Mokra D, Kopincova J, Kolomaznik M, Javorka M, Calkovska A (2017). Vagal-immune interactions involved in cholinergic anti-inflammatory pathway. Physiol Res.

[9] Bonaz Bruno, Sinniger Valérie, Pellissier Sonia (2018). Vagus Nerve Stimulation at the Interface of Brain–Gut Interactions. Cold Spring Harbor Perspectives in Medicine.

[10] Borovikova LV, Ivanova S, Zhang M, Yang H, Botchkina GI, Watkins LR, Wang H, Abumrad N, Eaton JW, Tracey KJ (2000). Vagus nerve stimulation attenuates the systemic inflammatory response to endotoxin. Nature.

[11] Johnson RL, Wilson CG (2018). A review of vagus nerve stimulaton as a therapeutic intervention. J Inflamm Res.

[12] Li Z, Liu X, Zhang Y, Shi J, Zhang Y, Xie P, Yu T (2014). Connection changes in somatosensory cortex induced by different doses of propofol. PLoS One.

[13] Lee SW, Kulkarni K, Annoni EM, Libbus I, KenKnight BH, Tolkacheva EG (2018). Stochastic vagus nerve stimulation affects acute heart rate dynamics in rats. PLoS One.

[14] Stauss HM (2017). Differential hemodynamic and respiratory responses to right and left cervical vagal nerve stimulation in rats. Physiol Rep.

[15] Broncel A, Bocian R, Kłos-Wojtczak P, Konopacki J (2018). Medial septal cholinergic mediation of hippocampal theta rhythm induced by vagal nerve stimulation. PLoS One.

[16] Stauss HM, Stangl H, Clark KC, Kwitek AE, Lira VA (2018). Cervical vagal nerve stimulation impairs glucose tolerance and suppresses insulin release in conscious rats. Physiol Rep.

[17] Cao J, Lu KH, Powley TL, Liu Z (2017). Vagal nerve stimulation triggers widespread responses and alters large-scale functional connectivity in the rat brain. PLoS One.

[18] Livak KJ, Schmittgen TD (2001). Analysis of relative gene expression data using real-time quantitative PCR and the 2(-Delta Delta C(T)) Method. Methods.

[19] Xin X, Xin F, Chen X, Zhang Q, Li Y, Huo S, Chang C, Wang Q (2017). Hypertonic saline for prevention of delirium in geriatric patients who underwent hip surgery. J Neuroinflammation.

[20] Nadelson MR, Sanders RD, Avidan MS (2014). Perioperative cognitive trajectory in adults. Br J Anaesth.

[21] Kalliolias GD, Ivashkiv LB (2016). TNF biology, pathogenic mechanisms and emerging therapeutic strategies. Nat Rev Rheumatol.

[22] Hua FZ, Ying J, Zhang J, Wang XF, Hu YH, Liang YP, Liu Q, Xu GH (2016). Naringenin pre-treatment inhibits neuroapoptosis and ameliorates cognitive impairment in rats exposed to isoflurane anesthesia by regulating the PI3/Akt/PTEN signalling pathway and suppressing NF-κB-mediated inflammation. Int J Mol Med.

[23] Zheng JW, Meng B, Li XY, Lu B, Wu GR, Chen JP (2017). NF-κB/P65 signaling pathway: a potential therapeutic target in postoperative cognitive dysfunction after sevoflurane anesthesia. Eur Rev Med Pharmacol Sci.

[24] Bonaz B, Sinniger V, Pellissier S (2016). Anti-inflammatory properties of the vagus nerve: potential therapeutic implications of vagus nerve stimulation. J Physiol.

[25] Huffman WJ, Subramaniyan S, Rodriguiz RM, Wetsel WC, Grill WM, Terrando N (2019). Modulation of neuroinflammation and memory dysfunction using percutaneous vagus nerve stimulation in mice. Brain Stimul.

[26] Maurer SV, Williams CL (2017). The Cholinergic System Modulates Memory and Hippocampal Plasticity via Its Interactions with Non-Neuronal Cells. Front Immunol.

[27] Zhou JP, Wang F, Li RL, Yuan BL, Guo YL (2004). Effects of febrile seizure on motor, behavior, spatial learning and memory in rats. Zhonghua Er Ke Za Zhi.

[28] Liu RP, Fang JL, Rong PJ, Zhao Y, Meng H, Ben H, Li L, Huang ZX, Li X, Ma YG, Zhu B (2013). Effects of electroacupuncture at auricular concha region on the depressive status of unpredictable chronic mild stress rat models. Evid Based Complement Alternat Med.

[29] Liu AF, Zhao FB, Wang J, Lu YF, Tian J, Zhao Y, Gao Y, Hu XJ, Liu XY, Tan J, Tian YL, Shi J (2016). Effects of vagus nerve stimulation on cognitive functioning in rats with cerebral ischemia reperfusion. J Transl Med.

[30] Zhu YZ, Yao R, Zhang Z, Xu H, Wang LW (2016). Parecoxib prevents early postoperative cognitive dysfunction in elderly patients undergoing total knee arthroplasty: A double-blind, randomized clinical consort study. Medicine (Baltimore).

[31] Chen K, Wei P, Zheng Q, Zhou J, Li J (2015). Neuroprotective effects of intravenous lidocaine on early postoperative cognitive dysfunction in elderly patients following spine surgery. Med Sci Monit.

[32] Pappa M, Theodosiadis N, Tsounis A, Sarafis P (2017). Pathogenesis and treatment of post-operative cognitive dysfunction. Electron Physician.

